# Common Variable Immunodeficiency Non-Infectious Disease Endotypes Redefined Using Unbiased Network Clustering in Large Electronic Datasets

**DOI:** 10.3389/fimmu.2017.01740

**Published:** 2018-01-09

**Authors:** Jocelyn R. Farmer, Mei-Sing Ong, Sara Barmettler, Lael M. Yonker, Ramsay Fuleihan, Kathleen E. Sullivan, Charlotte Cunningham-Rundles, The USIDNET Consortium, Jolan E. Walter

**Affiliations:** ^1^Massachusetts General Hospital, Boston, MA, United States; ^2^Department of Population Medicine, Harvard Pilgrim Health Care Institute, Harvard Medical School, Boston, MA, United States; ^3^Ann and Robert H. Lurie Children’s Hospital of Chicago, Chicago, IL, United States; ^4^Children’s Hospital of Philadelphia, Philadelphia, PA, United States; ^5^Icahn School of Medicine at Mount Sinai, New York, NY, United States; ^6^University of South Florida, St. Petersburg, FL, United States; ^7^Johns Hopkins All Children’s Hospital, St. Petersburg, FL, United States

**Keywords:** common variable immunodeficiency, non-infectious complications, endotypes, atopy, autoimmunity, lymphoproliferation, unbiased network clustering

## Abstract

Common variable immunodeficiency (CVID) is increasingly recognized for its association with autoimmune and inflammatory complications. Despite recent advances in immunophenotypic and genetic discovery, clinical care of CVID remains limited by our inability to accurately model risk for non-infectious disease development. Herein, we demonstrate the utility of unbiased network clustering as a novel method to analyze inter-relationships between non-infectious disease outcomes in CVID using databases at the United States Immunodeficiency Network (USIDNET), the centralized immunodeficiency registry of the United States, and Partners, a tertiary care network in Boston, MA, USA, with a shared electronic medical record amenable to natural language processing. Immunophenotypes were comparable in terms of native antibody deficiencies, low titer response to pneumococcus, and B cell maturation arrest. However, recorded non-infectious disease outcomes were more substantial in the Partners cohort across the spectrum of lymphoproliferation, cytopenias, autoimmunity, atopy, and malignancy. Using unbiased network clustering to analyze 34 non-infectious disease outcomes in the Partners cohort, we further identified unique patterns of lymphoproliferative (two clusters), autoimmune (two clusters), and atopic (one cluster) disease that were defined as CVID non-infectious endotypes according to discrete and non-overlapping immunophenotypes. Markers were both previously described {high serum IgE in the atopic cluster [odds ratio (OR) 6.5] and low class-switched memory B cells in the total lymphoproliferative cluster (OR 9.2)} and novel [low serum C3 in the total lymphoproliferative cluster (OR 5.1)]. Mortality risk in the Partners cohort was significantly associated with individual non-infectious disease outcomes as well as lymphoproliferative cluster 2, specifically (OR 5.9). In contrast, unbiased network clustering failed to associate known comorbidities in the adult USIDNET cohort. Together, these data suggest that unbiased network clustering can be used in CVID to redefine non-infectious disease inter-relationships; however, applicability may be limited to datasets well annotated through mechanisms such as natural language processing. The lymphoproliferative, autoimmune, and atopic Partners CVID endotypes herein described can be used moving forward to streamline genetic and biomarker discovery and to facilitate early screening and intervention in CVID patients at highest risk for autoimmune and inflammatory progression.

## Introduction

Common variable immunodeficiency (CVID) is the most frequent symptomatic primary immune deficiency worldwide (1, 2). The diagnosis requires low immunoglobulin levels (low IgG in combination with low IgA and/or low IgM for age-matched reference range), a demonstration of poor antibody-specific response to antigen challenge, and the exclusion of secondary causes. Historically, the clinical presentation of CVID centered around predisposition to recurrent infections; however, the definition has more recently expanded to include autoimmunity or lymphoproliferation as primary clinical presentations (1).

CVID epidemiology has been described almost exclusively at large national referral centers and centralized databases such as the United States Immunodeficiency Network (USIDNET). These data demonstrate the high prevalence of inflammatory and autoimmune complications in CVID, with non-infectious disease outcomes previously estimated at 68–74% and associated with up to an 11-fold increase in patient mortality (3–5). The national burden of CVID is also substantial and was recently estimated at US $274,200 per patient annually (6). Models for cost reduction include facilitating an earlier diagnosis as well as reducing rates of premature death in CVID patients with infiltrative lymphocytic complications, specifically (6, 7).

Substantial research effort over the last decade has centered around trying to identify the CVID subset that will develop inflammatory and autoimmune complications. To date, this research has primarily focused on specific non-infectious disease outcomes, including lymphadenopathy (LAD), splenomegaly, autoimmune cytopenias, and organ-specific lymphoproliferative disease (5, 8–17). The Freiburg (8), Paris (9), and EUROclass (10) trials were the first to use peripheral B cell flow cytometry to subcategorize CVID, which successfully identified low number of class-switched memory B cells as a marker for lymphoproliferative disease. More recently, defects in the T cell compartment including low total CD4+ T cells as well as reduced numbers of naïve CD4+ and CD8+ T cells have been associated with increased risk for specific autoimmune and lymphoproliferative complications including autoimmune cytopenias (13, 17), enteropathy (11, 17), and granulomatous-interstitial lung disease (GLILD) (15). Reclassification of this CVID subset as late-onset combined immunodeficiency has been proposed (11). Finally, lymphoproliferative pathology and progressive GLILD specifically have been correlated with high serum IgM (5, 14). However, an unbiased approach to categorizing the full spectrum of non-infectious disease complications seen in CVID has been lacking in the field. Moreover, CVID sub-classification using the Freiburg, Paris, or EUROclass criteria was recently demonstrated to be ineffective in determining underlying genetic etiology (18). These data suggest that more exact methods of non-infectious disease phenotyping in CVID are warranted to facilitate earlier diagnosis and targeted therapeutic intervention.

Recent advances in network theory have facilitated novel methods for studying biological structures and disease patterns (19–22). Unbiased network clustering can elucidate unique relationships between seemingly distinct pathophenotypes and has proof-of-principle benefit in the discovery of comorbidities when applied to human disease (23, 24). As CVID encompasses a heterogeneous clinical spectrum with now a growing list of diverse causative monogenic mutations (25), we hypothesized that network clustering may be uniquely applicable in discerning both convergent and divergent patterns of immunopathology that associate with risk for autoimmune and inflammatory progression.

Herein, we introduce the Partners CVID cohort as a well-annotated tertiary care dataset and perform comparative analysis with the USIDNET regarding immunophenotype and non-infectious disease outcomes. Using unbiased network clustering, we further identify unique patterns of lymphoproliferative, autoimmune, and atopic complications in the Partners cohort that are defined as CVID non-infectious disease endotypes according to discrete and non-overlapping immunophenotypes. These endotypes can be used moving forward to streamline genetic and biomarker discovery in order to improve non-infectious disease risk modeling in CVID.

## Materials and Methods

### CVID Cohort Assembly

The USIDNET is a program of the Immune Deficiency Foundation. The cohort was assembled by submitting a search query for “common variable immunodeficiency” to the USIDNET in 2016. This query yielded 918 patients. We excluded patients based on alternative primary diagnoses (combined immunodeficiency in 22 cases, transient hypogammagloblulinemia of infancy in 7 cases, hemophagocytic lymphohistiocytosis in 3 cases, autoimmune lymphoproliferative syndrome in 1 case, and x-linked lymphoproliferative disease subtype 2 in 1 case). This resulted in 884 patients in the USIDNET CVID cohort at the time of review.

The Partners CVID cohort was assembled following approval by the Partners Institutional Review Board with subsequent data collection and analysis performed according to the Institutional Review Board-approved protocol. A search query for ICD 9 and ICD 10 codes of “common variable immunodeficiency” in combination with low total IgG for age-matched reference range was submitted in 2013 to the Partners Research Patient Data Registry, which included clinical data from the Brigham and Women’s Hospital, the Massachusetts General Hospital, and the Massachusetts General Hospital for Children. This initial search query identified 411 patients with extractable data in the period of 2006–2013. In further chart review, only patients with a diagnosis of CVID by an immunologist or confirmatory laboratory evidence of CVID were further included, which resulted in 169 patients. From 2013–2017, new diagnoses of CVID presenting to the Partners Allergy/Immunology inpatient or outpatient practices were additionally included. This resulted in a total of 205 patients in the Partners CVID cohort at the time of review.

### Scoring of Non-Infectious Disease Outcomes

Non-infectious disease outcomes were scored as annotated in the USIDNET. For each patient in the Partners cohort, the complete electronic medical record (EMR) was searched across clinical notes, laboratory data, radiology, and pathology using the web-based natural language processing tool “Queriable Patient Inference Dossier” as previously described (26, 27). Extracted EMR data were then independently scored for non-infectious disease outcomes by direct physician review as detailed below.

#### End-Organ Infiltrative/Lymphoproliferative Disease

Granulomas and sarcoidosis were defined as biopsy-proven disease according to the interpreting pathologist. LAD and splenomegaly were defined as radiographic evidence of disease and were further subcategorized by biopsy as available. Celiac disease was defined by positive serology (anti-tissue transglutaminase antibodies) or biopsy-consistent disease according to the interpreting pathologist. GLILD was defined as either radiographic or biopsy-proven evidence of disease according to recent criteria (15, 28). On imaging, a chest computed tomography (CT) scan demonstrating bilateral ground-glass opacities or 4+ discrete nodules >1 mm in size was scored positive for GLILD (29). On either transbronchial or open lung biopsy, a report of granulomas, lymphocytic interstitial pneumonia, follicular bronchiolitis, and/or lymphoid hyperplasia was scored as positive for GLILD (30); this latter group of patients was further subcategorized as biopsy-proven GLILD. Autoimmune hepatitis was defined by liver injury in association with positive serology (anti-mitochondrial/smooth muscle antibodies) and/or biopsy-proven evidence of inflammatory pathology in the absence of active infection (infiltrating lymphocytes, granulomas, and/or regenerative fibrosis) as previously described for CVID (12, 31). Patients who met specific pathologic criteria for nodular regenerative hyperplasia (NRH) (12, 31) were further subcategorized. Autoimmune gastrointestinal disease was defined as chronic diarrhea with protein wasting and/or biopsy-proven evidence of immune pathology in the absence of active infection. Patients were further subcategorized as either autoimmune enteropathy (AIE) according to biopsy-proven evidence of cell dropout (e.g., parietal or goblet), villous blunting, increased intra-epithelial lymphocytosis, and/or follicular lymphoid hyperplasia, or as inflammatory bowel disease (IBD) according to biopsy-proven lymphoid aggregates and/or granulomas with a consideration of Crohn’s disease or ulcerative colitis in the differential diagnosis of the interpreting pathologist as previously described for the inflammatory gastrointestinal manifestations of CVID (32). Pulmonary hypertension (PH) was defined by positive history on physician chart review or otherwise as captured by review of all chest CT, echocardiogram, and right heart catheterization data in the Partners EMR according to adult criteria as pulmonary arterial dilation ≥2.9 cm on chest CT (33), right ventricular systolic pressure >30 mmHg on echocardiogram to capture the lower limit previously described (34), and/or mean pulmonary arterial pressure >25 mmHg at rest on right heart catheterization as available (35). Nasal polyps were defined by presence on CT scan, direct visualization on rhinoscopy, or biopsy-proven disease. When available, biopsy-proven disease was further subcategorized as eosinophilic or lymphoproliferative according to the interpreting pathologist. Thyroid nodules were defined as radiographic evidence of disease.

#### Cytopenias

Chronic anemia, thrombocytopenia, neutropenia, and lymphopenia were defined by positive history on physician chart review or otherwise as captured by review of all cytology in the Partners EMR as total hemoglobin, platelet count, absolute neutrophil count, or absolute lymphocyte count less than age-matched reference range and persistent over >12 months. Chronic cytopenias that were presumed iatrogenic or infectious were specifically excluded. Cytopenias were further subcategorized as autoimmune hemolytic anemia (AIHA), immune thrombocytopenia (ITP), or autoimmune neutropenia (AIN) according to a presumed diagnosis of exclusion or positive autoantibodies (Coombs, anti-platelet and/or -neutrophil) as previously described (36–38).

#### Autoimmune Disease

Psoriasis, alopecia, vitiligo, and chronic intermittent urticaria were defined by positive history on physician chart review. Inflammatory arthritis was defined as a chart history of rheumatoid arthritis (positive rheumatoid factor or anti-cyclic citrullinated peptide antibodies), seronegative inflammatory arthritis, juvenile idiopathic arthritis, psoriatic arthritis, or inflammatory arthritis not otherwise specified with specific exclusion of isolated arthralgias and osteoarthritis. Autoimmune neuropathy was defined as a chart history of autoimmune motor or sensory loss and included the diagnoses of non-infectious uveitis, Bell’s Palsy, myasthenia gravis, multiple sclerosis, and biopsy-confirmed peripheral small fiber polyneuropathy. Autoimmune thyroid disease was defined as hypothyroidism requiring hormone replacement and was further subcategorized as Hashimoto’s thyroiditis (positive anti-thyroperoxidase antibodies). Bronchiectasis was defined as radiographic evidence of disease.

#### Atopic Disease

Asthma, eczema, and allergic rhinitis were defined by positive history on physician chart review. Asthma and eczema were further subcategorized by Partners subspecialty care (allergy, pulmonology, and/or dermatology) and disease severity (e.g., intermittent, mild, moderate, or severe). Allergic rhinitis was further subcategorized by environmental allergen skin testing results as available.

#### Malignancy

Hematologic malignancy was defined as biopsy-proven leukemia, lymphoma, myelodysplasia, multiple myeloma, and/or mastocytosis according to the interpreting pathologist. Solid organ cancer was defined by positive history on physician chart review and was further subcategorized by anatomic location.

### Scoring of Immunophenotype

Immunophenotype was scored by direct physician review of the EMR for each patient in the Partners cohort or as annotated in the USIDNET as follows: native immunoglobulin levels (IgG, IgA, IgM, IgE, and IgG subclasses 1–4), IgG replacement level (median IgG while receiving immunoglobulin replacement therapy), total lymphocytes (CD3+, CD4+, CD8+, CD3− CD56+ CD16+, and CD19+ as absolute counts on peripheral flow cytometry), B cell maturation (CD27+ and CD27+ IgD− as percent of CD19+ B cells on peripheral flow cytometry), T cell maturation (CD45RA+ and CD45RO+ as percent of CD4+ cells on peripheral flow cytometry), complement levels (C3, C4, and CH50), pneumococcal titers [post-vaccination with 23-valent pneumococcal polysaccharide vaccine as available as percent protective (≥1.3 μg/mL) per serotypes tested], T cell proliferation to mitogen (percent of CD3+ response to phytohemagglutinin and pokeweed) and antigen (percent of CD3+ response to *Candida* and tetanus antigens).

### Unbiased Network Clustering Analysis

A data-driven network approach was used to derive disease endotypes based on the presenting complications and the associated immunophenotypes in independent cohorts: Partners (*n* = 205), USIDNET adult (*n* = 571), or USIDNET pediatric (*n* = 212). Accordingly, a network was constructed to model the inter-relationships among disease complications. Schematically, the model was represented as a graph, with nodes representing disease complications, and links between two nodes denoting statistically significant correlations between two disease complications, quantified using chi-square statistic. In order to minimize uncertainties in our analysis, we excluded weaker correlations with an odds ratio (OR) of less than three in the Partners cohort or less than two in the USIDNET and analyzed disease etiologies represented in less than 2% of the cohort as a larger group (e.g., systemic autoantibody disease). The network was then partitioned into subgraphs comprising highly connected nodes using the Girvan–Newman clustering algorithm (20). These subgraphs represent combinations of CVID complications with a high likelihood of co-occurrence. In the Partners cohort, patients were further assigned to individual network-derived clusters if they had two or more of the disease complications captured within that cluster. We then identified immunophenotypes that were significantly associated with each cluster and the association of each cluster with mortality using chi-square statistic. The term “endotype” was applied to any CVID cluster defined by a distinct subset of immune markers to suggest a unique immunopathology. The term “pathophenotype” herein refers to a disease subset where the clinical presentation has been linked causally with an underlying disease mechanism.

### Statistical Analysis

Comparative statistical analyses between cohorts were conducted using a two-tailed Student’s *t* test. Statistical analyses defining association within the Partners cohort were conducted using a chi-square test. In all analyses, a *P* value of <0.05 was considered significant.

## Results

### Comparative Demographics and Immunophenotypes

**Figure 1:**
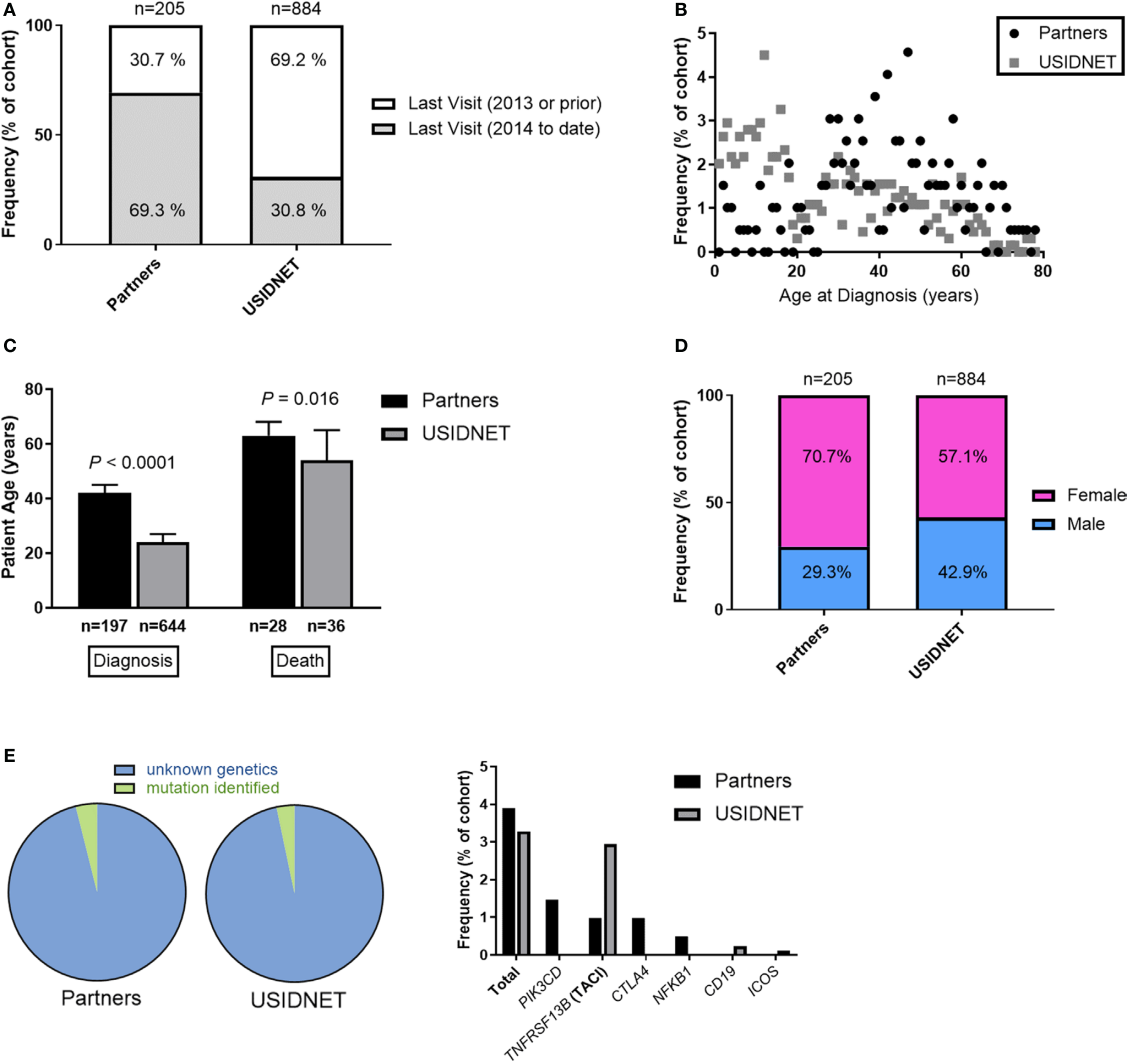
Comparative demographics between the Partners and United States Immunodeficiency Network (USIDNET) common variable immunodeficiency (CVID) cohorts. **(A)** Active patient follow-up, shown as percentage of total cohort. **(B)** Patient age at time of diagnosis, shown as frequency by age. **(C)** Patient age at time of diagnosis or death, shown as median ±95% CI with statistical significance indicated by *P* value. **(D)** Gender distribution, shown as percentage of total cohort. **(E)** Patients with an identified mutation in a CVID-associated gene, shown as percentage of total cohort.

In the USIDNET cohort, 272 patients (30.8%) were recently seen at an entering institution (2014–to date) whereas 612 patients (69.2%) had a more remote visit entered in the system (1988–2013). By comparison in the Partners cohort, 142 patients (69.3%) had a recent visit in the Partners EMR (2014–to date) whereas 63 patients (30.7%) were seen more remotely at a Partners institution (1998–2013) (Figure 1A). In comparison to the USIDNET, there was a significantly older median age at time of diagnosis in the Partners cohort (Figures 1B,C, 42 vs. 24 years, *P* < 0.0001), which likely reflects the high capture of pediatric patients with primary immune deficiency at Boston Children’s Hospital (outside of the Partners network hospitals). Given the limitation in active follow-up and the larger fraction of pediatric cases in the USIDNET cohort, only 36 patients (4.1%) were annotated as deceased at a median of 54 years (range 25–80 years) compared to 31 patients (15.1%) at a median of 63 years (range 41–83 years) in the Partners cohort at the time of review, which met statistical significance for older age at time of death in the Partners cohort (Figure 1C, *P* = 0.016). Gender demonstrated a female predominance in both cohorts, however, was increased comparatively in the Partners cohort at 70.7 vs. 57.1% female (Figure 1D, *P* = 0.00034). Finally, patients with an identified underlying CVID-associated genetic mutation were extremely limited in both cohorts (3.9% in Partners vs. 3.3% in USIDNET) with exact etiologies as shown (Figure 1E).

**Figure 2:**
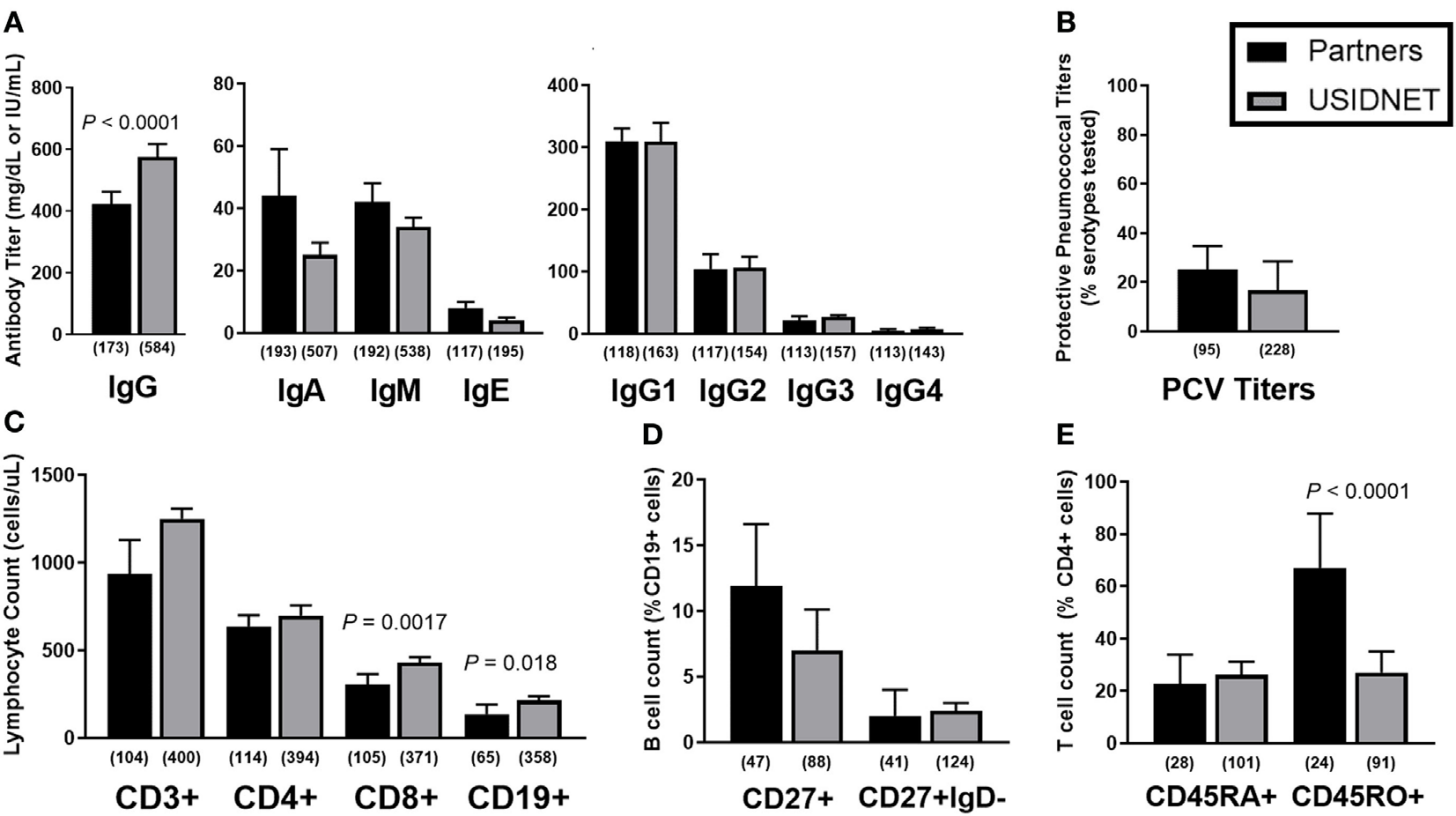
Comparative immunophenotypes between the Partners and United States Immunodeficiency Network (USIDNET) common variable immunodeficiency (CVID) cohorts. **(A)** Native immunoglobulin levels, **(B)** protective pneumococcal vaccine (PCV) titers, **(C)** total lymphocyte counts, **(D)** B cell maturation, and **(E)** T cell maturation shown as median ± 95% CI. Number of patients reported per immune parameter shown in parentheses. Statistical significance indicated by *P* value.

Immunophenotypes were highly comparable between the two cohorts. Native immunoglobulin levels demonstrated slightly lower IgG in the Partners cohort (Figure 2A, median 422 vs. 575 mg/dL, *P* < 0.0001) but otherwise similar deficiencies were identified in IgA (median 44 vs. 25 mg/dL), IgM (median 42 vs. 34 mg/dL), IgE (median 8 vs. 4 IU/mL), and IgG subclasses 1–4 (median IgG1 309.5 vs. 309, IgG2 104 vs. 106.5, IgG3 21 vs. 27, and IgG4 4.6 vs. 7 mg/dL). These trends persisted despite extraction of the USIDNET pediatric population (immunoglobulin levels obtained ≤18 years of age) (Figure S1A in Supplementary Material). Protective titers to pneumococcus were defined as ≥1.3 μg/mL in the Partners cohort or otherwise as annotated in the USIDNET. Calculated percentages of protective titers per pneumococcal serotypes tested were then compared across cohorts as previously described for the diagnostic criteria of CVID (1). This analysis demonstrated similar impairment in antibody response to pneumococcus in both cohorts (Figure 2B, median 25.0 vs. 16.7%). Finally, lymphocyte subsets were analyzed in both cohorts (Partners vs. USIDNET). Absolute counts of total CD3+ T cells (median 935.5 vs. 1,249.25 cells/μL) and CD4+ T cells (median 630.5 vs. 698 cells/μL) were not statistically different. However, absolute counts of CD8+ T cells (median 303 vs. 431 cells/μL, *P* = 0.0017) and CD19+ B cells (median 124 vs. 214 cells/μL, *P* = 0.018) were significantly lower in the Partners cohort (Figure 2C). With further extraction of the USIDNET pediatric population (lymphocyte counts obtained ≤18 years of age), this difference in CD19+ B cells resolved (median adult count 180 cells/μL, *P* = 0.16) and the difference in CD8+ T cells trended toward similar (median adult count 376.5 cells/μL, *P* = 0.030) (Figure S1B in Supplementary Material), suggesting that increased pediatric cases contributed in part to the observation of higher total lymphocyte counts in the USIDNET. Flow cytometry assessment of B and T cell maturation was limited in both cohorts (CD27+ IgD− staining in CD19+ B cells annotated in 20.0% Partners vs. 14.0% USIDNET; CD45RA+ staining in CD4+ T cells annotated in 13.7% Partners vs. 11.4% USIDNET). Given these limitations, a similar degree of maturation arrest at the level of memory (median 11.9 vs. 7.0% CD27+) and class-switched memory (median 2.0 vs. 2.4% CD27+ IgD−) was observed in the CD19+ B cell compartment (Figure 2D). In comparison, a similar reduction in naïve CD4+ T cell counts (median 22.75 vs. 26.1% CD45RA+) but with a preferential expansion in memory CD4+ T cell counts (median 67.0 vs. 27.0% CD45RO+, *P* < 0.0001) was observed in the Partners cohort (Figure 2E). This CD45RO+ CD4+ T cell expansion persisted in the Partners cohort despite extraction of the USIDNET pediatric population, which demonstrated an expected trend toward increased naïve and decreased memory CD4+ T cells (median adult count 34.0% CD45RO+, *P* = 0.00035) (Figure S1D in Supplementary Material).

**Figure 3:**
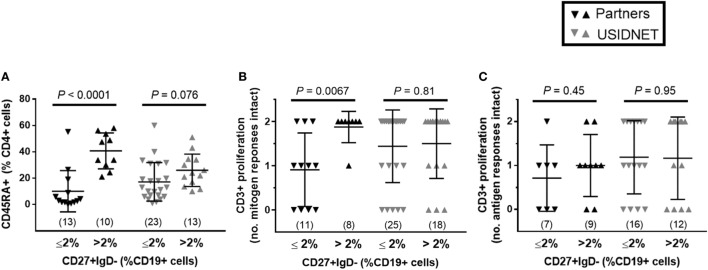
Overlapping B and T cell immunopathologies in the Partners and United States Immunodeficiency Network (USIDNET) common variable immunodeficiency (CVID) cohorts. Comparison by class-switched memory B cell severity (CD27+ IgD− ≤2% vs. >2% CD19+ cells) of **(A)** naïve CD4+ T cell counts (CD45RA+ cells shown as percent of CD4+ cells), **(B)** CD3+ T cell proliferation to mitogen (number (no.) of intact responses to phytohemagglutinin and pokeweed stimulation shown), and **(C)** CD3+ T cell proliferation to antigen [number (no.) of intact responses to *Candida* and tetanus stimulation shown]. Symbols denote individual patients; black bars denote median ± 95% CI. Number of patients reported per immune parameter shown in parentheses. Statistical significance indicated by *P* value.

To further identify potential late-onset combined immunodeficiency cases (11), low class-switched memory B cell counts (CD27+ IgD− ≤2% CD19+ cells) were used as a marker of severe B cell immunopathology as previously described for CVID (10) and assayed for overlapping defects in the CD4+ T cell compartment. In both cohorts, there was a trend toward lower naïve CD4+ T cells in patients with ≤2% class-switched memory B cells, which met statistical significance in the Partners cohort (*P* < 0.0001, Figure 3A). Additional analysis of CD3+ T cell proliferation to *in vitro* mitogen or antigen challenge demonstrated a preferential defect in patients with ≤2% class-switched memory B cells following mitogen challenge in the Partners cohort (*P* = 0.0067, Figure 3B), whereas no difference was observed following mitogen challenge in the USIDNET cohort or following antigen challenge in either cohort (Figures 3B,C).

Together these data confirmed that in comparison to the national CVID registry (USIDNET), the Partners cohort had similar if not more severe immunopathology (lower native IgG levels, lower CD8+ and CD19+ lymphocyte counts, and increased shift toward memory CD4+ T cells), due in part to an older patient demographic. Furthermore, while lack of routine B and T cell immunophenotyping prohibited an accurate assessment of combined immunodeficiency frequency in both cohorts, the overlapping reduction in naïve CD4+ T cell counts and response to mitogen challenge in patients with ≤2% class-switched memory B cells in the Partners cohort was consistent with the identification of a late-onset combined immunodeficiency phenotype.

### Comparative Non-Infectious Disease Outcomes

**Figure 4:**
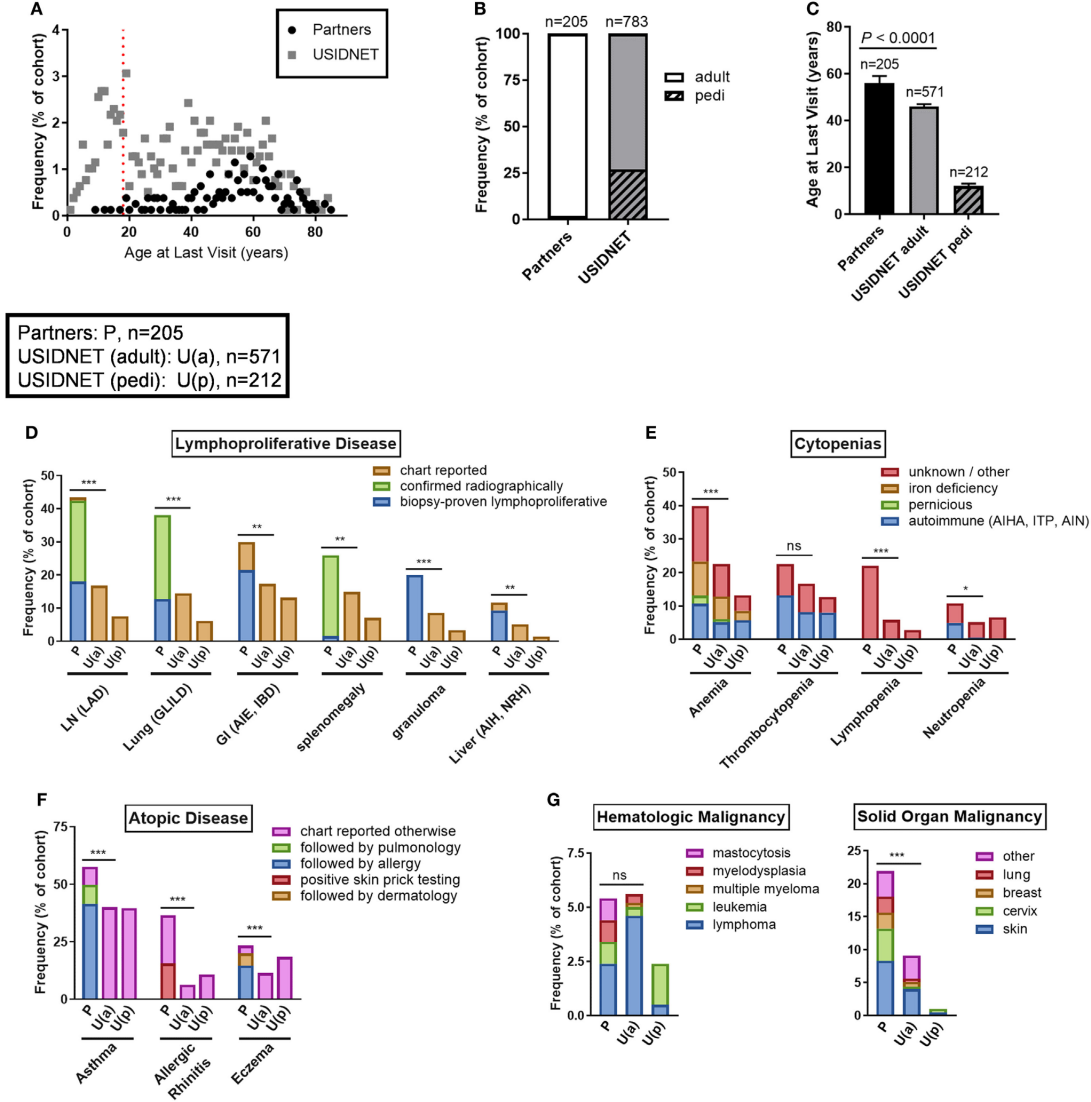

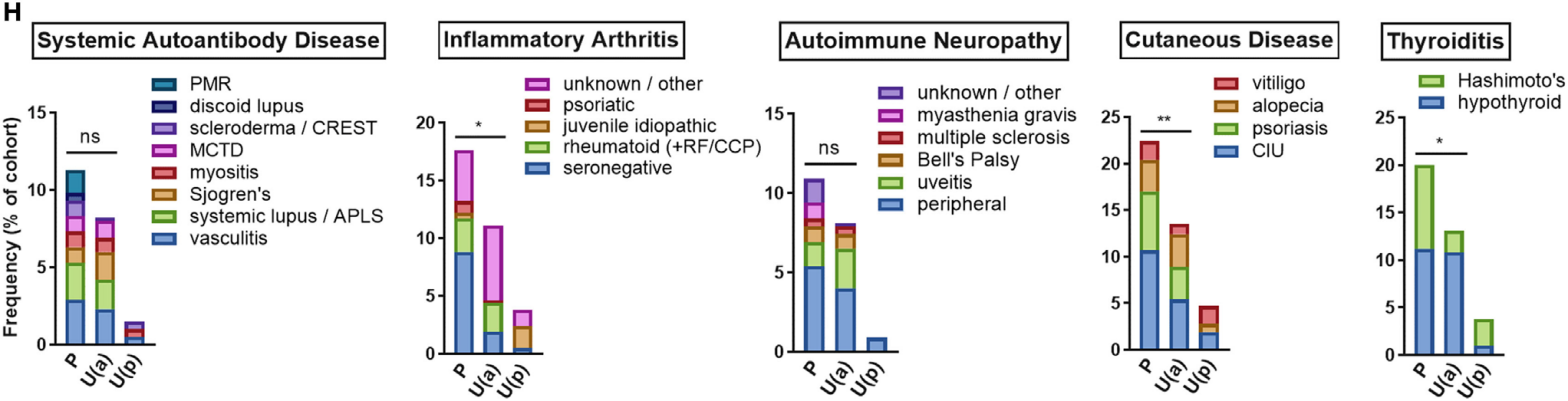
Comparative non-infectious disease complication rates between the Partners and United States Immunodeficiency Network (USIDNET) common variable immunodeficiency (CVID) cohorts. **(A)** Patient age at time of last visit, shown as frequency by age with dotted red line indicating the pediatric cutoff (≤18 years). **(B)** Adult vs. pediatric distribution at time of last visit, shown as percentage of total cohort. **(C)** Patient age at time of last visit, shown as median ± 95% CI with statistical significance indicated by *P* value. Frequency of **(D)** lymphoproliferative disease, **(E)** cytopenias, **(F)** atopic disease, **(G)** malignancy, and **(H)** autoimmunity, shown as percentage of total cohort with available data entry [Partners: P, *n* = 205; USIDNET adult: U(a), *n* = 571, USIDNET pediatric: U(p), *n* = 212]. Statistical significance between the Partners total and USIDNET adult cohorts is indicated for the total outcome represented in the bar (ns = not statistically different, *P < 0.05, **P < 0.005, ***P < 0.0001). AIE, autoimmune enteropathy; AIH, autoimmune hepatitis; AIHA, autoimmune hemolytic anemia; AIN, autoimmune neutropenia; APLS, antiphospholipid syndrome; CCP, cyclic citrullinated peptide; CIU, chronic intermittent urticaria; GI, gastrointestinal; GLILD, granulomatous-interstitial lung disease; IBD, inflammatory bowel disease; ITP, immune thrombocytopenia; LAD, lymphadenopathy; LN, lymph node; MCTD, mixed connective tissue disease; NRH, nodular regenerative hyperplasia; pedi, pediatric; PMR, polymyalgia rheumatica; RF, rheumatoid factor.

Non-infectious disease outcomes were annotated for all 205 patients in the Partners cohort compared to only 783 patients who had data entry for this field in the USIDNET. As the Partners cohort was an older demographic at the time of diagnosis with a higher percentage of active patient follow-up, only four patients remained in the pediatric category (≤18 years of age) at the time of last recorded visit to a Partners institution, consistent with a median age of 56 years at last follow-up in the Partners cohort (Figures 4A–C). In contrast, 27.1% of patients in the USIDNET remained in the pediatric category (≤18 years of age) at the time of last recorded visit (Figures 4A,B). Therefore, we chose to analyze non-infectious disease outcomes separately in the USIDNET adult (*n* = 571, median age of 46 years at last follow-up) and USIDNET pediatric (*n* = 212, median age of 12 at last follow-up) cohorts (Figure 4C). Moreover, as accumulation of non-infectious disease complications may be age-dependent (3), statistical comparisons were drawn only between the Partners and USIDNET adult cohorts.

Comparable to increased non-infectious disease rates were observed in the Partners cohort across all complications analyzed including end-organ lymphoproliferative disease, cytopenias, atopy, malignancy, and autoimmunity (Figures 4D–H). End-organ lymphoproliferative disease was further subcategorized in the Partners cohort by disease confirmed radiographically or by biopsy demonstrating benign lymphoproliferative pathology. Specific chart review of all radiology in the Partners EMR for these complications likely contributed to a high capture rate, however, even when limited to biopsy-proven disease the Partners cohort had similar rates of LAD (18.0 vs. 16.8%, *P* = 0.69), GLILD (12.7 vs. 14.5%, *P* = 0.51), and autoimmune gastrointestinal disease including AIE and IBD (21.5 vs. 17.3%, *P* = 0.18) and increased rates of granulomas (20.0 vs. 8.6%, *P* < 0.0001) and non-infectious hepatitis including autoimmune hepatitis and NRH (9.3 vs. 5.1%, *P* = 0.033) as compared to the USIDNET adult cohort (Figure 4D; Table S1 in Supplementary Material). Chronic cytopenias were also comparable to increased in the Partners cohort (Figure 4E; Table S2 in Supplementary Material). This included autoimmune cytopenias specifically: AIHA (10.7 vs. 5.1%, *P* = 0.0051), ITP (13.2 vs. 8.1%, *P* = 0.032), and AIN (4.9 vs. 0.5, *P* < 0.0001). Here again, chart review of all cytology in the Partners EMR likely contributed to a high capture rate. Atopic disease was increased overall in the Partners cohort, with increased rates to the USIDNET adult population even when limited to asthma receiving Partners subspecialty care (49.8 vs. 40.1%, *P* = 0.017), eczema receiving Partners subspecialty care (19.5 vs. 11.4%, *P* = 0.0036), or allergic rhinitis with confirmed positive skin testing (15.6 vs. 6.3, *P* < 0.0001) (Figure 4F; Table S3 in Supplementary Material). Hematologic malignancies were comparable between adult cohorts including similar frequencies of lymphoma (2.4 vs. 4.6%, *P* = 0.18) and leukemia (1.0 vs. 0.4%, *P* = 0.29) (Figure 4G; Table S4 in Supplementary Material). In contrast, solid organ malignancy was increased in the Partners cohort with major sites including the skin, cervix, breast, and lung (Figure 4G; Table S4 in Supplementary Material). Finally, autoimmunity was comparable to increased in the Partners cohort across the spectrum of systemic autoantibody disease, inflammatory arthritis, autoimmune neuropathy, cutaneous disease, and thyroiditis (Figure 4H; Table S5 in Supplementary Material). Breakdown to individual disease etiologies revealed increased frequency in the Partners cohort as follows: polymyalgia rheumatica (1.5 vs. 0.0%, *P* = 0.0038), seronegative arthritis (8.8 vs. 1.9%, *P* < 0.0001), myasthenia gravis (1.0 vs. 0.0%, *P* = 0.018), chronic intermittent urticaria (10.7 vs. 5.4%, *P* = 0.0010), and Hashimoto’s thyroiditis (8.8 vs. 2.3%, *P* < 0.0001) (Figure 4H, Table S5 in Supplementary Material).

### Non-Infectious Disease Endotypes in the Partners Cohort

**Figure 5:**
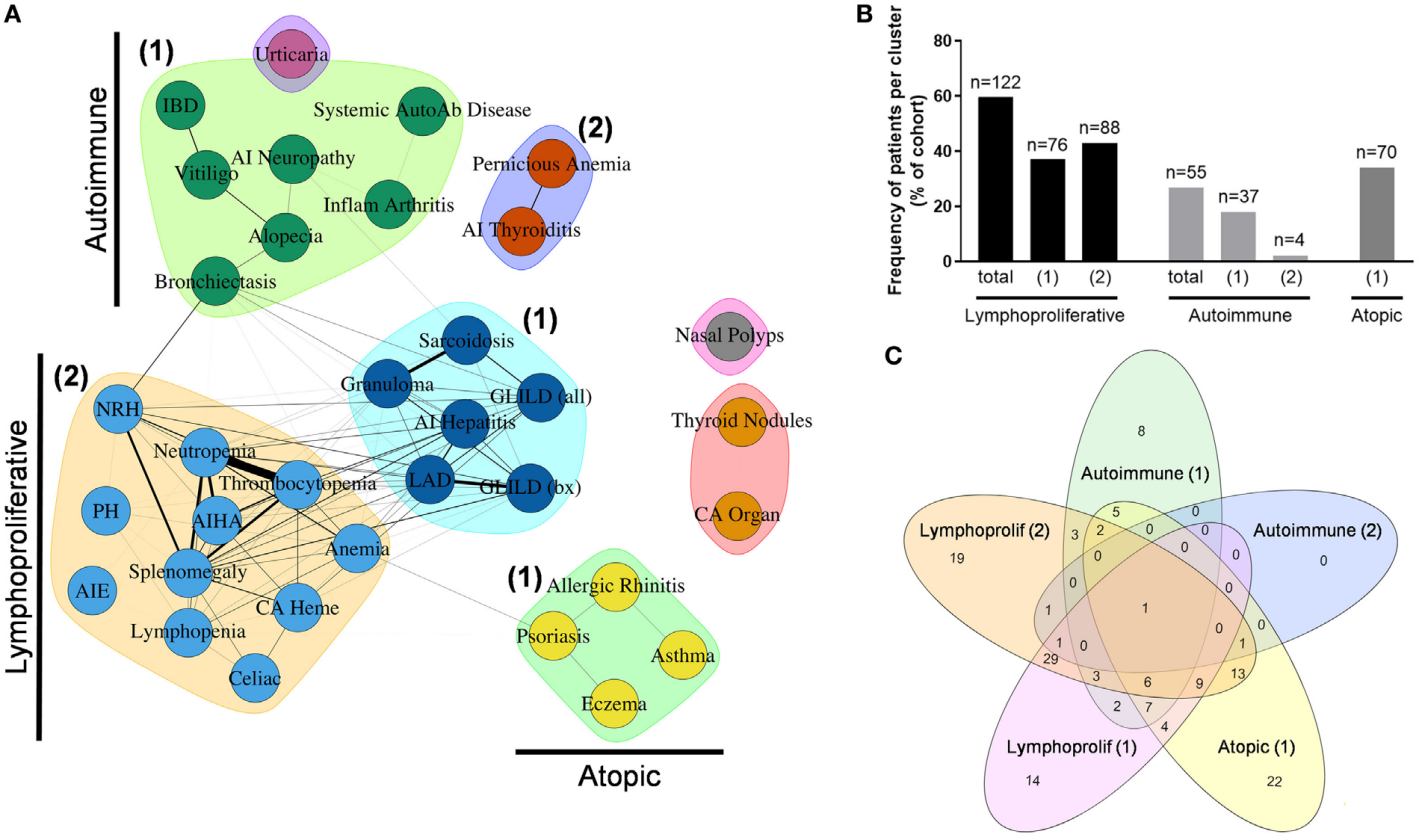
Unbiased network clustering of non-infectious disease complications in the Partners cohort. **(A)** Graph of inter-relationship among 34 non-infectious disease complications in the Partners cohort defined using unbiased network clustering. Nodes in the graph represent disease complications; links between nodes denote statistically significant relations between comorbidities; clustering of comorbidities represents disease patterns with high likelihood of co-occurrence as defined by the Girvan–Newman clustering algorithm (20). The weight of each network link correlates with the strength of the association between two comorbidities, as measured by chi-square test (*P* < 0.05). Nodes in isolation indicate disease complications that failed to cluster due to a lack of association with other comorbidities. Subgraphs are annotated as lymphoproliferative (1 and 2), autoimmune (1 and 2), and atopic (1) clusters, respectively. **(B)** Patient assignment by non-infectious disease cluster, shown as percentage of total cohort. **(C)** Cluster overlap, shown as a Venn diagram assembled using the web-based tool InteractiVenn as previously described (43). AI, autoimmune; AIE, autoimmune enteropathy; AIHA, autoimmune hemolytic anemia; autoab, auto-antibody; bx, biopsy-proven; CA heme, hematologic cancers; CA organ, solid organ cancers; chronic intermittent urticaria (urticaria); GLILD, granulomatous-interstitial lung disease; IBD, inflammatory bowel disease; LAD, lymphadenopathy; NRH, nodular regenerative hyperplasia; PH, pulmonary hypertension.

To further determine inter-relationships between the non-infectious outcomes identified in the Partners cohort, we used unbiased network clustering. This analysis yielded discrete clusters that were predominantly lymphoproliferative (2 clusters), autoimmune (2 clusters), and atopic (1 cluster) in etiology (Figure 5A). Notable exceptions included localization of psoriasis to the atopic cluster, which may represent diagnostic uncertainty with eczema in this condition, as well as localization of bronchiectasis and IBD to autoimmune cluster 1, which may represent unanticipated autoimmune co-occurrence with these conditions (39, 40) and a divergence of immunopathology between IBD and AIE (41, 42). In contrast, localization of cytopenias and hematologic malignancy to lymphoproliferative cluster 2 is consistent with previously described coincidence of autoimmune cytopenias and lymphoproliferative pathology in CVID (10). Finally, solid organ malignancy and thyroid nodules formed an independent cluster, and nasal polyps were not associated with any other outcome analyzed, which may represent dual pathologies of eosinophilic and lymphocytic polyposis contained within this broader categorization (Table S1 in Supplementary Material).

Next, patients were assigned to individual clusters by definition of having two or more outcomes contained within that cluster. This analysis revealed a significant burden of non-infectious disease across the Partners cohort with 122 patients (59.5%) contained within the total lymphoproliferative cluster, 55 patients (26.8%) contained within the total autoimmune cluster, and 70 patients (34.1%) contained within the atopic cluster (Figure 5B). In contrast, 44 patients (21.5%) failed to localize to a non-infectious disease cluster, consistent with an infections only phenotype as previously reported for CVID (3–5). Finally, analysis of patient overlap demonstrated cluster assignment to be highly specific with a maximum of 49 patients (23.9%) overlapping between lymphoproliferative clusters 1 and 2 (Figure 5C).

To determine whether cluster assignment correlated with immunophenotype, OR association with cluster assignment was analyzed across 25 different immune parameters. This analysis revealed immunophenotypic correlations that were specific to the lymphoproliferative, autoimmune, and atopic clusters, respectively (Table 1). Consistent across all lymphoproliferative clusters (1, 2, and in total) was an association with low class-switched memory B cells (CD27+ IgD− ≤2% CD19+ cells), low total CD19+ B cells (<90 cell/μL), and loss of naïve CD4+ T cells (CD45RA+ <20% and CD45RO+ >70% CD4+ cells). Immunopathology associated with lymphoproliferative cluster 2 specifically that additionally carried over to the lymphoproliferative cluster in total included CD4+ T cell lymphopenia (<419 cell/μL), high serum IgM (>334 mg/dL), and low C3 levels (<93 mg/dL). These data both confirmed and expanded on previously defined associations with lymphoproliferative pathology in CVID [low class-switched memory B cells (10), low total B cells (3), high serum IgM (5, 14), and skewed CD4+ T cell maturation (11, 13, 15, 17)] as well as defined a novel association (low C3). In contrast, none of the 25 immune parameters analyzed were found to be significantly associated with the autoimmune clusters (1, 2, or in total), and only high serum IgE (>100 IU/mL) significantly associated with the atopic cluster, consistent with previous description of high serum IgE as a marker of atopic disease (44, 45). In summary, these data demonstrated both validated and novel immunophenotypic correlations that were highly cluster-specific and entirely non-overlapping, consistent with the identification of distinct lymphoproliferative, autoimmune, and atopic CVID non-infectious disease endotypes through the use of unbiased network clustering.

**Table 1:** Immunophenotype correlation with cluster assignment in the Partners cohort.

Immune parameter (reference range)	Lymphoproliferative			Autoimmune			Atopic
	Cluster (1)	Cluster (2)	Cluster (total)	Cluster (1)	Cluster (2)	Cluster (total)	Cluster (1)
IgG (614–1,295 mg/dL)							
IgA (69–309 mg/dL)							
IgM (53–334 mg/dL)		1.9* (1.03–3.4)/1.1* (1.02–1.14)		1.1* (1.01–1.1)			
IgE (0–100 IU/mL)							6.5** (1.7–24.9)
IgG1 (382.4–928.6 mg/dL)							
IgG2 (241.8–700.3 mg/dL)							
IgG3 (21.8–176.1 mg/dL)							
IgG4 (3.9–86.4 mg/dL)							
IgG (median on replacement therapy) (800–1,400 mg/dL)							
CD3+ (690–2,540 cells/μL)		5.3*** (2.0–13.9)					
CD4+ (419–1590 cells/μL)		6.8*** (2.5–18.1)	4.7** (1.5–14.7)				
CD8+ (190–1,140 cells/μL)		2.6* (1.05–6.6)					
CD16/56+ CD3− (90–590 cells/μL)							
CD19+ (90–660 cells/μL)	3.4* (1.3–8.6)	3.8* (1.4–9.8)	3.8* (1.3–11.0)				
CD27+ (5–25% of CD19+)							
CD27+ IgD− (2–20% of CD19+)	7.3* (1.6–33.1)	12.8*** (2.9–56.6)	9.2** (2.0–41.7)				
CD45RA+ (20–70% of CD4+)	23.8*** (3.4–169.4)	3.8*** (1.8–8.1)	3.3*** (1.7–6.5)				
CD45RO+ (20–70% of CD4+)	9.8* (1.6–61.6)	2.8** (1.5–5.4)	2.6* (1.4–4.6)				
PHA proliferation (≥58.5% of CD3+)							
PWM proliferation (≥3.5% of CD3+)							
*Candida* proliferation (≥3.0% of CD3+)							
Tetanus proliferation (≥3.3% of CD3+)							
C3 (93–202 mg/dL)		11.2*** (2.9–43.3)	5.1* (1.1–24.0)				
C4 (16–41 mg/dL)		6.1* (1.5–24.0)					
CH50 (63–145 mg/dL)							

Statistically significant correlations are shown as odds ratio with 95% confidence interval, *P < 0.05, **P < 0.005, ***P < 0.0001. Blue/red indicates an association with low/high immune parameter levels, respectively.

*PHA, phytohemagglutinin; PWM, pokeweed mitogen*.

Finally, unbiased network clustering of non-infectious disease complications was attempted in the USIDNET dataset. In the pediatric cohort (*n* = 212), this analysis yielded discrete atopic and lymphoproliferative clusters (Figure S2A in Supplementary Material). Of note in this population, the autoimmune cytopenias were strongly associated and clustered independently, and there was an absence of the total autoimmune disease cluster previously identified in the Partners cohort, likely driven by the low frequency of these complications overall in the pediatric population (Figure 4H). In contrast, there was a lack of association between known comorbidities in the USIDNET adult cohort (*n* = 571) (Figure S2B in Supplementary Material). We were, therefore, unable to derive meaningful network clusters of non-infectious disease complications for the adult population.

### Mortality Risk in the Partners Cohort

Risk of death in the Partners cohort was analyzed across all non-infectious disease outcomes and immunophenotypes independently as well as within the lymphoproliferative, autoimmune, and atopic clusters identified. For the individual non-infectious disease outcomes, mortality was most strongly associated with lymphopenia (OR 12.9, *P* < 0.0001), PH (OR 6.6, *P* < 0.0001), AIHA (OR 4.0, *P* = 0.001), and autoimmune hepatitis (OR 3.0, *P* = 0.040) in addition to thrombocytopenia, solid organ malignancy, splenomegaly, and LAD (Table 2, top panel). Analysis of the individual immunophenotypes further delineated an association with CD3+ (OR 4.6, *P* = 0.046) and CD4+ (OR 4.5, *P* = 0.017) T cell lymphopenia in addition to low C3 (<93 mg/dL, OR 6.5, *P* = 0.015) and low median IgG while receiving replacement immunoglobulin therapy (<800 mg/dL, OR 4.3, *P* = 0.004) (Table 2, middle panel). Finally, among the non-infectious disease endotypes identified in the Partners cohort, risk of death was specifically associated with lymphoproliferative cluster 2 (OR 5.9, *P* < 0.0001) (Table 2, bottom panel).

**Table 2:** Mortality risk in the Partners cohort.

	*N*	Odds ratio (95% CI)	*P*-value
**Non-infectious disease outcome**			
Lymphopenia	21	12.9 (5.4–30.6)	<0.0001
Pulmonary hypertension	13	6.6 (2.8–15.7)	<0.0001
Autoimmune hemolytic anemia	16	4.0 (1.8–8.9)	0.001
Autoimmune hepatitis	8	3.0 (1.2–7.6)	0.04
Thrombocytopenia	13	2.9 (1.3–6.6)	0.014
Splenomegaly	14	2.8 (1.3–6.2)	0.017
Solid organ cancer	12	2.8 (1.2–6.3)	0.024
Lymphadenopathy	19	2.4 (1.1–5.3)	0.04
**Immunophenotype**			
Low C3 (<93 mg/dL)	6	6.5 (1.6–26.2)	0.015
Low CD3+ T cells (<690 cells/μL)	6	4.6 (1.2–17.9)	0.045
Low CD4+ T cells (<419 cells/μL)	8	4.5 (1.4–14.2)	0.017
Low median IgG replacement level (<800 mg/dL)	16	4.3 (1.6–11.1)	0.004
**Cluster**			
Lymphoproliferative cluster 2	24	5.9 (2.4–14.4)	<0.0001

*All statistically significant positive correlations shown as number of deceased patients (*N*), odds ratio (OR), 95% confidence interval (95% CI), and *P* value*.

## Discussion

Primary immune deficiency disorders remain low frequency in the general population with an estimated prevalence for CVID of 1:10,000 to 1:100,000 (1). Therefore, primary immune deficiency disease epidemiology benefits greatly from the assembly of national cohorts and databases such as the USIDNET. Our data importantly highlight the applicability of these national data in validating results obtained from smaller, regional cohorts. Here, we demonstrate the Partners CVID cohort to be a slightly older age demographic with increased female predominance as compared to the USIDNET, however, with comparable immunophenotype as measured by native immunoglobulin levels, titer response to pneumococcus, and B cell maturation arrest at the memory and class-switched memory stages. The lower absolute lymphocyte counts identified in the Partners cohort, including CD19+ B cells and CD8+ T cells, were due in part to age-related changes in an older patient demographic (46, 47). However, we cannot exclude an additional, potentially bidirectional, association between the higher non-infectious disease complication rates and more severe immunopathology identified in the Partners cohort. In both CVID cohorts, there was a reduction in naïve CD4+ T cell counts with a trend toward overlapping lower class-switched memory B cell counts that met statistical significance in the Partners cohort. CD4+ T cell dysfunction, as measured by decreased proliferation to *in vitro* mitogen stimulation, also overlapped with lower class-switched memory B cell counts in the Partners cohort. These data suggest the identification of a late-onset combined immunodeficiency phenotype, preferentially in the Partners cohort, as previously described for CVID (11). However, routine clinical B and T cell maturation phenotyping will be necessary moving forward to obtain a more accurate frequency of late-onset combined immunodeficiency cases.

Limitations inherent to data entry at the national level include lack of capture, lack of active follow-up, and non-uniform field entry, all of which potentially contributed to lower non-infectious disease complication rates in the USIDNET as compared to previous single-center analysis within the United States (3). The power of the Partners cohort included active follow-up in 69.3% of patients, a shared EMR amenable to web-based searches using natural language processing, and direct physician scoring of each non-infectious disease complication. Additionally, while Partners network hospitals are a non-national referral center for CVID, they are renowned national institutions with likely referral bias for the secondary non-infectious disease complications herein described. Finally, despite restricting non-infectious disease rate comparisons to the USIDNET adult population only, there was a persistent older median age at time of last visit in the Partners cohort (56 vs. 46 years, *P* < 0.0001). All of these factors likely contributed to the higher capture rate of non-infectious disease complications in the Partners cohort, best exemplified by the frequency of unanticipated complications that were identified, including PH and solid organ malignancy (14.6 and 22.0% in the Partners cohort, respectively). The occurrence of PH in primary immune deficiency has been limited previously to case reports (48, 49) and was annotated at only 0.5% in the USIDNET adult cohort. Solid organ malignancy has been reported at only 5–7% in CVID cohorts previously (3, 4) and was annotated at only 9.1% in the USIDNET adult cohort. Furthermore, both outcomes were independently associated with increased risk of death in the Partners cohort (OR 6.6 and 2.8, respectively). Together these data suggest that PH and solid organ malignancy, including skin and cervical cancer as the major etiologies identified here, may be underrecognized complications in CVID. Consistent with these data, recent retrospective analysis of 1,583 pediatric PH cases identified an unexpected association with primary immune deficiency (OR 37.9) (50). Further detailed analyzes are underway in the Partners cohort to determine the underlying pathophysiology and importantly, whether additional screening is required for these complications in the CVID demographic moving forward. However, the applicability of these findings may be limited to large tertiary care centers within the United States.

Unbiased network clustering has the novel potential to decipher patterns of inter-relationship among seemingly distinct pathophenotypes in human disease (23, 24) and, therefore, may have unique applicability in genetically heterogeneous clinical entities like CVID, where risk modeling for non-infectious disease development remains elusive despite more than a decade of subcategorization by more traditional methods (5, 8–17). Within the Partners CVID cohort, unbiased network clustering successfully converged 34 non-infectious disease complications into discrete lymphoproliferative, autoimmune, and atopic patterns as well as diverged non-infectious disease complications that might otherwise have been clinically misclassified (localizing AIHA outside of the autoimmune cluster, for example). Furthermore, cluster assignment was demonstrated to be specific (≤23.9% overlap between any two clusters) and associated with unique immunophenotypic markers consistent with CVID non-infectious disease endotypes. One limitation to the unbiased network clustering method used in this analysis, however, was the definition of association using an OR of three. While a high OR was specifically chosen to limit spurious correlations in a small sample size, it could limit discovery of more subtle disease inter-relationships. Therefore, the non-infectious disease correlations identified in the Partners cohort are by definition strongly associated but not exhaustive. In contrast, there was a lack of association between known comorbidities in the adult USIDNET population, which prevented the formation of a meaningful cluster network. We hypothesize that this lack of association between known comorbidities may be driven by comorbidity under-reporting, as we identified lower non-infectious disease complication rates in the USIDNET as compared to previous single-center analysis within the United States (3), or less accurate categorization, as we identified a high frequency of nonspecific diagnoses in the USIDNET (e.g., elevated liver function tests and chronic diarrhea) (Table S1 in Supplementary Material). As with any data-driven approach, the quality of network-based analyses is heavily dependent on the completeness and accuracy of the input data. Finally, in this study we searched the Partners EMR using natural language processing followed by direct physician scoring of the extracted data, which favors accuracy but is inherently time consuming. An alternative approach is to apply computational techniques, including machine learning and automated natural language processing, to streamline the process of phenotype abstraction from medical charts and network construction. Recent studies have demonstrated the feasibility of using such approaches to identify phenotypic patterns captured in EMRs, and their potential in expediting discovery research (51, 52). There is further potential in applying computational approaches to integrate data from multiple sources to drive phenotype-genotype research.

Unbiased network clustering in the Partners cohort identified known as well as novel immunophenotype-non-infectious disease phenotype correlations. Low class-switched memory B cell counts (CD27+ IgD− ≤2% CD19+ cells) were strongly associated with all lymphoproliferative clusters as previously described (10). Low naïve CD4+ T cell counts, which previously have been associated with CVID-related autoimmune cytopenias and end-organ lymphoproliferative pathology (11, 13, 17), additionally associated with all lymphoproliferative clusters in the Partners cohort. The correlation between lymphoproliferative cluster 1 and low CD45RA+ (OR 23.8)/high CD45RO+ (OR 9.8) was particularly impressive and higher in magnitude than the cluster-specific correlation with low class-switched memory B cells (OR 7.3). Recently, decreased T cell receptor repertoire diversity was proposed as a primary mechanism of immunopathology in CVID patients with low class-switched memory B cells (53). Furthermore, the unmasking of monogenic causes of CVID including *CTLA4* and *PIK3CD* have demonstrated substantial lymphoproliferative disease burden in the context of chronic T cell activation through decreased regulatory cell inhibition or enhanced intracellular mTOR signaling, respectively (54–57). These data suggest that loss of naïve CD4+ T cells and skewing of the repertoire is a prominent and perhaps primary immunopathology in CVID-related lymphoproliferative disease development, consistent with a CVID subset that is enriched for late-onset combined immunodeficiency. Our data additionally highlight the potential utility of low CD45RA+ (<20% CD4+ cells) in subcategorizing CVID patients at risk for lymphoproliferative complications including LAD, granulomas, autoimmune hepatitis, and GLILD. Furthermore, as CVID classification by B cell phenotype alone was recently demonstrated to be ineffective in determining underlying genetic etiology (18), genotyping within the Partners cohort to determine whether non-infectious disease endotype can predict genetic etiology will be of high utility in the CVID field moving forward. Finally, as the Partners cohort is actively followed, naïve CD4+ T cell counts and receptor repertoire can be trended over clinical course to determine whether loss co-occurs with or rather predates, and thus predicts, lymphoproliferative disease development in CVID.

The additional observation of low complement levels in association with lymphoproliferative cluster 2 (C3, OR 11.2 and C4, OR 6.1) as well as with the lymphoproliferative cluster in total (C3, OR 5.1) was unexpected and not to our knowledge described previously. In contrast, increased levels of C3 and C4 were identified in a total CVID cohort comprised of 71 patients, 41% with autoimmune disease and 13% with granulomatous disease, as compared to 30 healthy controls (58). Together these data suggest that low serum levels of C3 and C4 may be uniquely useful in subcategorizing CVID patients at risk for non-infectious complications including AIE, cytopenias (AIHA), and NRH. Autoantibodies are known to directly mediate the pathology of autoimmune cytopenias, which can include classical complement activation in AIHA (59), ITP (60), and AIN (61). More recently, autoantibodies have been described in AIE (42) and NRH (62), although their role in disease pathogenesis is less clear. In CVID specifically, a break in central B cell tolerance driving autoantibody production has been described (63) and correlated with underlying genetic etiology in the case of monoallelic *TNFRS13B* (encoding TACI) mutations (64). As lymphoproliferative cluster 2 and low C3 levels were independently correlated with increased risk of death in the Partners cohort (OR 5.9 and 6.5, respectively), these data suggest a clinically important immunopathologic distinction. Further research is necessary in the Partners cohort to determine whether the immunopathology in lymphoproliferative cluster 2 converges at a break in B cell tolerance and/or is defined by underlying genetic risk.

Finally, in contrast to the lymphoproliferative and atopic correlations identified, none of the examined immune parameters significantly correlated with the autoimmune clusters (1, 2, or in total). This finding suggests a CVID subset with significant pathology that is poorly immunophenotyped by the currently available routine clinical parameters. Therefore, screening for novel biomarker associations within this autoimmune subset, specifically, would be of high utility in the CVID field moving forward.

## Author Contributions

JF wrote the manuscript, performed Partners chart review and the comparative cohort analyses; M-SO performed unbiased network clustering and related statistical analyses as well as provided statistical expertise; SB performed Partners chart review; LY performed initial 2013 Partners query; RF, KS, CC-R, and the USIDNET consortium contributed the CVID dataset, and JW conceived of the project and provided expertise in immunology.

## Conflict of Interest Statement

This research was supported in part by a publication research grant from CSL-Behring and an unrestricted scientific grant from Shire to the USIDNET, a program of the Immune Deficiency Foundation funded by the National Institute of Allergy and Infectious Diseases at the National Institutes of Health.

## Abbreviations

AIE, autoimmune enteropathy; AIHA, autoimmune hemolytic anemia; AIN, autoimmune neutropenia; CVID, common variable immunodeficiency; EMR, electronic medical record, GLILD, granulomatous-interstitial lung disease; IBD, inflammatory bowel disease; ITP, immune thrombocytopenia; LAD, lymphadenopathy; NRH, nodular regenerative hyperplasia; PH, pulmonary hypertension; USIDNET, United States Immunodeficiency Network.
