# Supplementary material for: Common Variable Immunodeficiency Non-Infectious Disease Endotypes Redefined Using Unbiased Network Clustering in Large Electronic Datasets
Source: Front Immunol. 2018 Jan 9;8:1740. doi: 10.3389/fimmu.2017.01740 (PMC5767273; doi:10.3389/fimmu.2017.01740)
Supplement: Supplementary file 1 [file Table_1.docx]

Supplementary Material

**Common Variable Immunodeficiency Non-Infectious Disease Endotypes Redefined Using Unbiased Network Clustering in Large Electronic Datasets**

Jocelyn R. Farmer*, Mei-Sing Ong, Sara Barmettler, Lael M. Yonker, Ramsay Fuleihan, Kathleen E. Sullivan, Charlotte Cunningham-Rundles, The USIDNET Consortium and Jolan E. Walter

*** Correspondence:** Jocelyn R. Farmer: jrfarmer@partners.org

# Supplementary Table 1: Comparative rates of end-organ infiltrative/lymphoproliferative disease between the Partners and USIDNET CVID cohorts

Frequency of non-infectious disease outcomes shown as total patient number (no.) with percentage of total cohort shown in parentheses. Comparative *P* value between Partners total and USIDNET adult cohorts is shown in black for each outcome (or in grey for the Partners qualified outcome vs. the USIDNET total outcome). Statistical significance indicated, **P* < 0.05, ** *P* < 0.005, *** *P* < 0.0001.


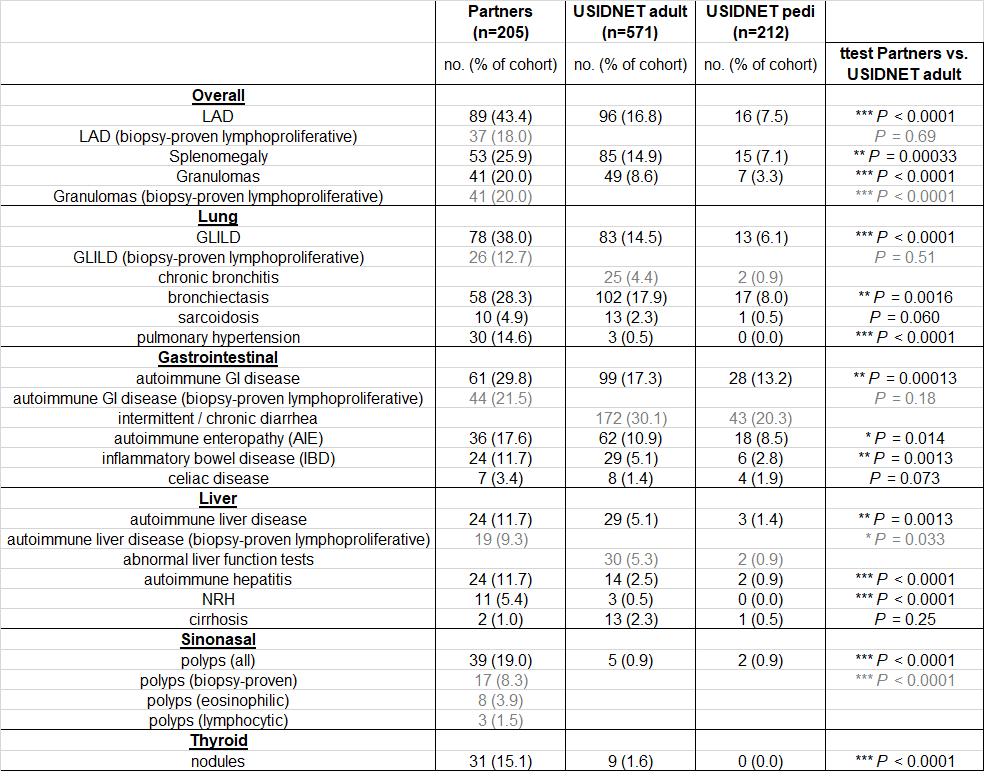


# Supplementary Table 2: Comparative rates of cytopenias between the Partners and USIDNET CVID cohorts

Frequency of non-infectious disease outcomes shown as total patient number (no.) with percentage of total cohort shown in parentheses. Comparative *P* value between Partners total and USIDNET adult cohorts is shown in black for each outcome. Statistical significance indicated, **P* < 0.05, ** *P* < 0.005, *** *P* < 0.0001. AIHA, autoimmune hemolytic anemia; AIN, autoimmune neutropenia; ITP, immune thrombocytopenia.


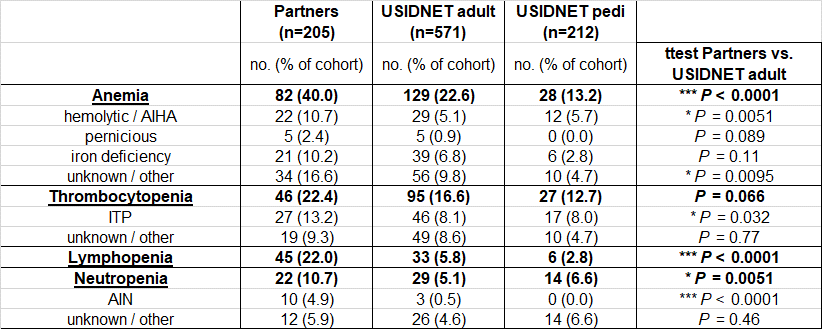


# Supplementary Table 3: Comparative rates of atopic disease between the Partners and USIDNET CVID cohorts

Frequency of non-infectious disease outcomes shown as total patient number (no.) with percentage of total cohort shown in parentheses. Comparative *P* value between Partners total and USIDNET adult cohorts is shown in black for each outcome (or in grey for the Partners qualified outcome vs. the USIDNET total outcome). Statistical significance indicated, **P* < 0.05, ** *P* < 0.005, *** *P* < 0.0001.


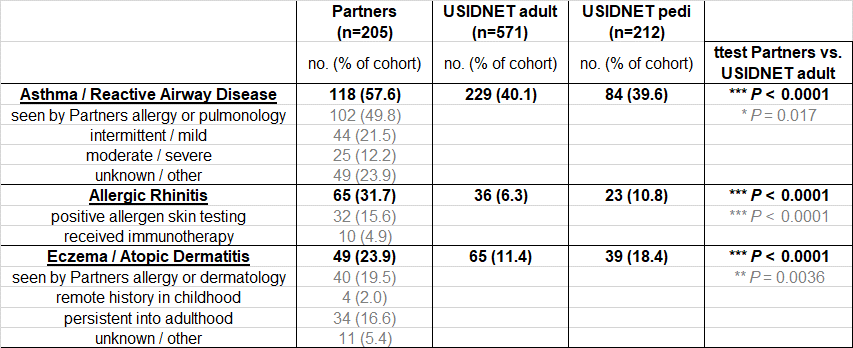


# Supplementary Table 4: Comparative rates of malignancy between the Partners and USIDNET CVID cohorts

Frequency of non-infectious disease outcomes shown as total patient number (no.) with percentage of total cohort shown in parentheses. Comparative *P* value between Partners total and USIDNET adult cohorts is shown in black for each outcome. Statistical significance indicated, **P* < 0.05, ** *P* < 0.005, *** *P* < 0.0001.


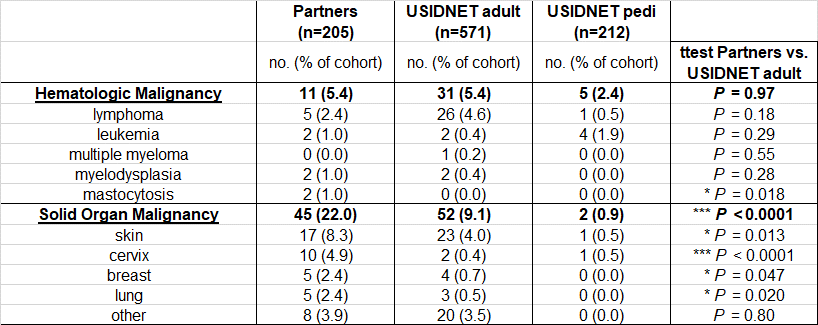


# Supplementary Table 5: Comparative rates of autoimmune disease between the Partners and USIDNET CVID cohorts

Frequency of non-infectious disease outcomes shown as total patient number (no.) with percentage of total cohort shown in parentheses. Comparative *P* value between Partners total and USIDNET adult cohorts is shown in black for each outcome. Statistical significance indicated, **P* < 0.05, ** *P* < 0.005, *** *P* < 0.0001. CCP, cyclic citrullinated peptide; RF, rheumatoid factor; TPO, thyroperoxidase.


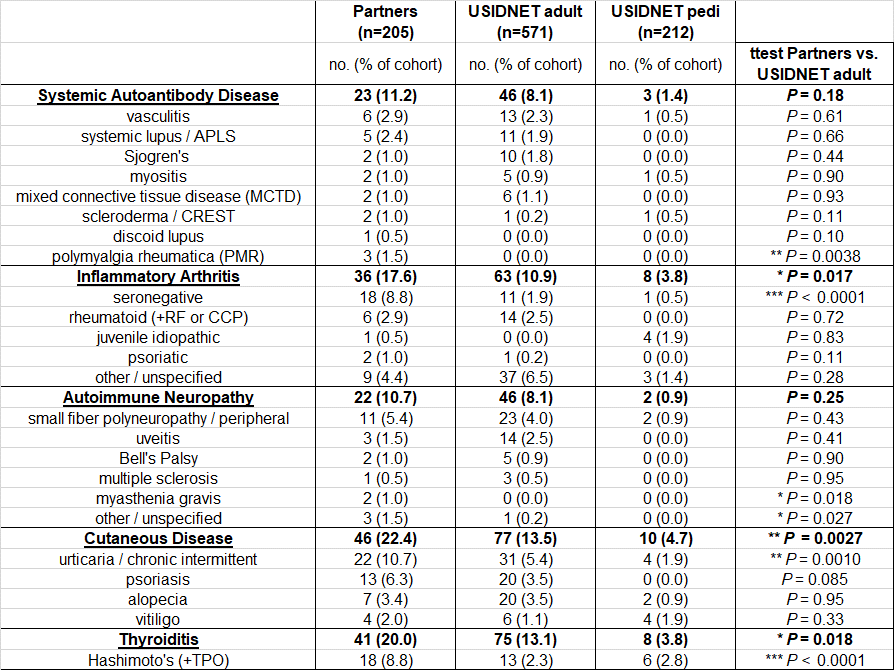


# Supplemental Figure 1. Comparative immunophenotypes between the Partners and USIDNET CVID cohorts with breakout of USIDNET pediatric patients

# (A) Native immunoglobulin levels, (B) total lymphocyte counts, (C) B cell maturation, and (D) T cell maturation shown as median +/- 95% CI. Number of patients reported per immune parameter shown in parentheses. Statistical significance indicated in comparison to the Partners cohort (ns = not statistically different, **P* < 0.05, ** *P* < 0.005, *** *P* < 0.0001).

#
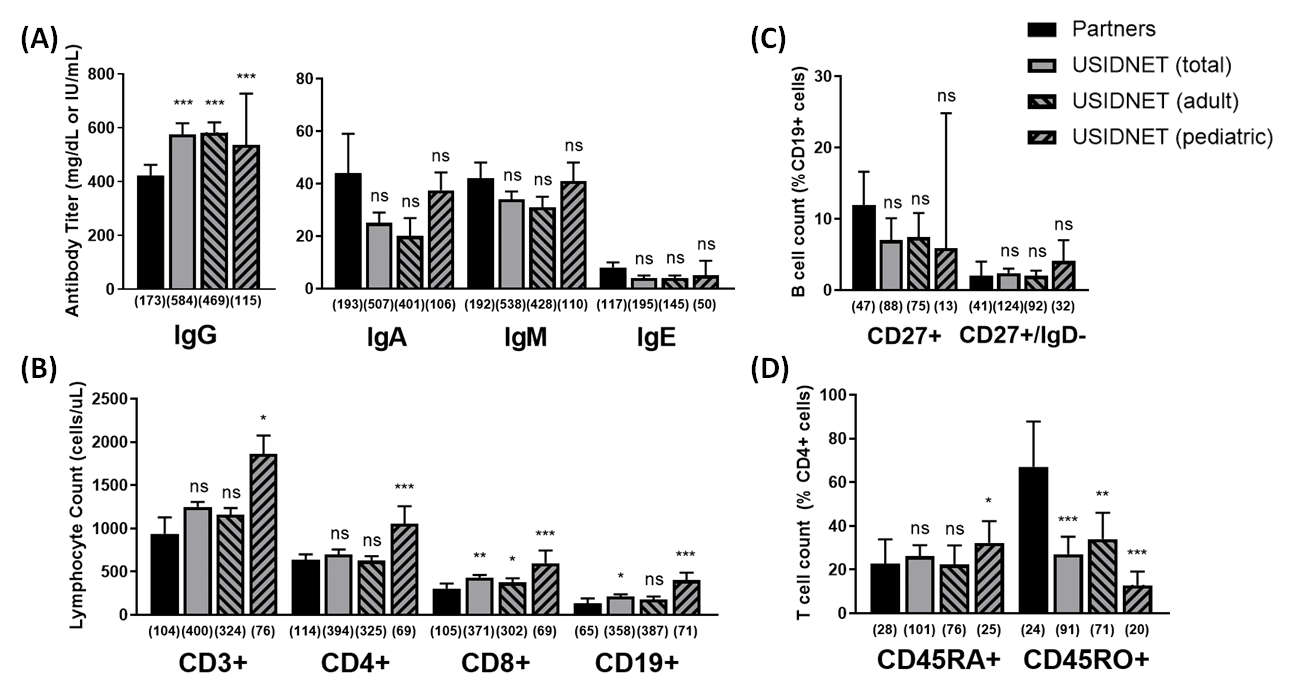


# Supplemental Figure 2. Unbiased network clustering of non-infectious disease complications in the USIDNET cohort

# (A) Graph of inter-relationship among non-infectious disease complications annotated at > 2% in the USIDNET pediatric cohort (n=212) defined using unbiased network clustering. Nodes in the graph represent disease complications; links between nodes denote statistically significant relations between comorbidities; clustering of comorbidities represents disease patterns with high likelihood of co-occurrence as defined by the Girvan-Newman clustering algorithm (20). The weight of each network link correlates with the strength of the association between two comorbidities, as measured by chi-square test (*P* < 0.05). AIE, autoimmune enteropathy; AIHA, autoimmune hemolytic anemia; AI thyroiditis, autoimmune thyroiditis; CA heme, hematologic cancers; CIU, chronic intermittent urticaria; GLILD, granulomatous-interstitial lung disease; Iron Def Anemia, iron deficiency anemia; ITP, immune thrombocytopenia; LAD, lymphadenopathy. (B) Lack of association between atopic known comorbidities in the USIDNET adult cohort (n=571), shown as paired non-infectious disease complications with corresponding odds ratio (OR), 95% CI, and *P* value indicated.

**
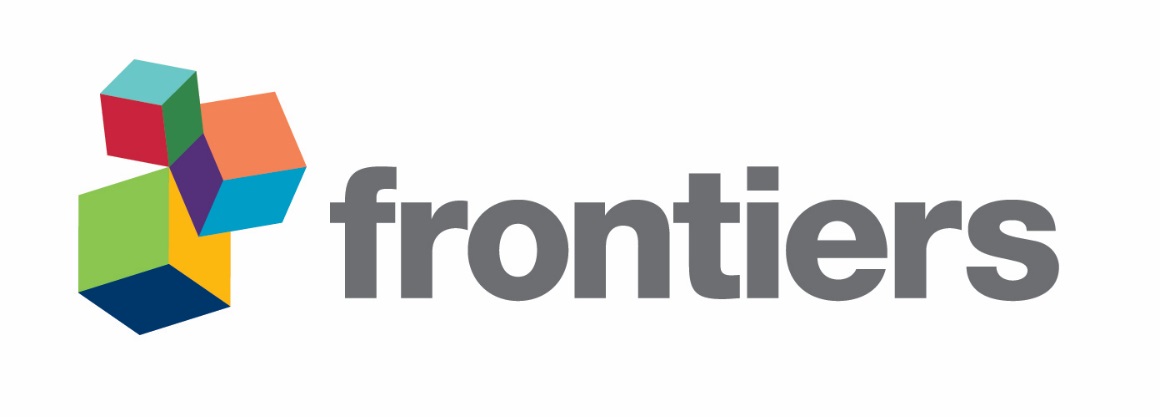
**
